# Human milk and breastfeeding at the first oral feed for infants with critical congenital heart disease: a multi-institutional study

**DOI:** 10.1038/s41372-026-02724-8

**Published:** 2026-05-26

**Authors:** Kristin M. Elgersma, Caelah Clark, Nancy L. Slater, Kimberly M. Morris, Mallory Moor, Adam Kortis, Chetna Pande, Karli A. Negrin, Samantha C. Butler

**Affiliations:** 1 School of Nursing, University of Minnesota, Minneapolis, MN USA; 2 Department of Therapy and Rehabilitative Services, Children’s Hospital Colorado, Aurora, CO USA; 3 Children’s Minnesota, Minneapolis, MN USA; 4 Rady Children’s Hospital San Diego, San Diego, CA USA; 5 James M. Anderson Center for Health Systems Excellence, Cincinnati Children’s Hospital Medical Center, Cincinnati, OH USA; 6 Department of Pediatrics, Baylor College of Medicine, Houston, TX USA; 7 Division of Critical Care Medicine, Texas Children’s Hospital, Houston, TX USA; 8 Department of Therapy and Rehabilitative Services, Nemours Children’s Health, Wilmington, DE USA; 9 Department of Psychiatry and Behavioral Sciences, Boston Children’s Hospital, Boston, USA; 10 Department of Psychiatry, Harvard Medical School, Boston, MA USA

**Keywords:** Epidemiology, Congenital heart defects

## Abstract

**Objective:**

Describe nutrition type/route at the first oral feed (FOF) for infants with critical congenital heart disease (CCHD); identify supportive/limiting factors.

**Study design:**

Retrospective cohort; adjusted regression estimated associations between parental/clinical factors and human milk or breastfeeding at the FOF.

**Result:**

For 1355 infants across 15 sites, human milk was used in 78.5% of FOFs, with 34.5% breastfeeding. Human milk was associated with parent presence (OR: 5.32, *p* < 0.001) and with feeding/lactation consults (2.49, *p* < 0.001). Private insurance predicted human milk (1.87, *p* < 0.001) and breastfeeding (1.82, *p* < 0.001). Younger age (<2 days; 2.23, *p* < 0.001) and preoperative status (3.66, *p* < 0.001) were associated with breastfeeding.

**Conclusion:**

This is the first description of human milk/breastfeeding at the FOF in CCHD. Parent presence and feeding/lactation support were potentially modifiable factors.

## Introduction

Infants who are hospitalized for congenital heart disease (CCHD) frequently experience delayed oral feeding initiation due to medical and surgical interventions for critical illness, clinical comorbidities, poor arousal and state regulation, and parent-infant separation during the postnatal period [1, 2]. While early feeding exposure for infants with CHD is associated with reduced likelihood of a feeding tube requirement at hospital discharge [3, 4], persistent feeding difficulties with dependence on alternative feeding routes (e.g., feeding tube, intravenous nutrition) may contribute to longer hospital length of stay and higher healthcare utilization [5]. For parents, feeding is a core relational activity that supports bonding, parental role attainment, and confidence in caring for their medically fragile infant [6, 7]. Moreover, breastfeeding (BF) initiation, an intricate neurodevelopmental process that depends on reciprocal learning between infant and parent [8], is often disrupted, which further constraints opportunities for parent-infant synchrony during this critical developmental window.

Human milk and direct BF provide the optimal, biologically normative nutrition for all infants and are unequivocally recommended for hospitalized infants due to disease reduction and neurodevelopmental benefit [9, 10]. For infants with CCHD, human milk feeding has been associated with substantial reductions in necrotizing enterocolitis and hospital length of stay, while direct BF has been associated with 93% lower sepsis and fewer adverse gastrointestinal events [11–14]. Importantly, human milk and BF are strongly preferred by most parents, including those of infants with CCHD (>90% in one CCHD study) [15]. Early feeding experiences play a critical role in shaping long-term nutrition for hospitalized infants, including those with CCHD [16–21]. One study of nearly 2500 infants with single ventricle CHD found that any BF prior to the stage 1 palliative surgery was associated with a twofold higher likelihood of receiving human milk, both at discharge and at the time of the stage 2 palliation nearly 5 months later, while any preoperative bottle feeding with commercial formula predicted lower human milk use [22]. Similarly, a single-site study of newborns undergoing surgery found that human milk use during the first enteral feed was associated with a 44% higher proportion of human milk intake during hospitalization and greater likelihood of human milk at discharge [23].

The first oral feed (FOF), defined as the first occasion an infant receives nutrition orally, is strongly related to human milk and BF duration [18, 19, 24, 25]. For preterm infants, direct BF at the FOF has been associated with higher rates of human milk feeding and BF throughout hospitalization and at discharge [19, 24], and a 2021 Cochrane review concluded that avoiding bottle use during preterm oral feeding initiation may increase BF rates for at least 6 months post-discharge [25]. This evidence suggests that, for hospitalized infants, the FOF may support maternal-infant exposure to the life course benefits of human milk and BF. Little is known, however, about the FOF specifically for infants with CCHD. To address this knowledge gap, we aimed to describe the nutrition type (maternal or donor human milk, commercial formula) and route (BF, bottle feeding) at the FOF for infants with CCHD across 15 geographically and institutionally diverse sites, and to identify differences in nutrition type and route based on key parental and clinical factors.

## Materials/subjects and methods

### Study design and setting

This retrospective cohort study represents a secondary analysis of data originating from a multisite quality improvement (QI) initiative focused on increasing parental engagement during the FOF for infants with CCHD. The QI initiative was conducted in 15 pediatric cardiac centers participating in the Cardiac Newborn Neuroprotective Network, a Special Interest Group of the Cardiac Neurodevelopmental Outcome Collaborative and supported by Cardiac Networks United. The initiative was grounded in a QI framework that included development of a key driver diagram highlighting family-centered education, staff awareness, workflow integration, and access to feeding specialists as primary drivers of improved parent involvement during the FOF. Boston Children’s Hospital Institutional Review Board approved this study and deemed it exempt (IRB-P00047550).

### Data collection

Data were abstracted retrospectively from electronic medical records using a standardized REDCap tool. A multidisciplinary team composed of physicians, nurses, feeding specialists, and QI experts created a comprehensive data dictionary before data collection. Abstractors were dedicated research assistants or the site principal investigator. All abstractors completed structured training and calibration to ensure consistent application of variable definitions and standardized data entry procedures. Data collection occurred from September 2022 through April 2025 and represented data collected throughout the QI project. Data were deidentified at the site level before being uploaded to the central REDCap database. A data analyst supporting the initiative conducted routine data quality checks throughout the project, including monthly review and generation of control charts to identify inconsistencies, missing data, or deviations from expected patterns. Sites were contacted for clarification or correction when data irregularities were identified.

### Participants

Inclusion criteria were infants diagnosed with CCHD, defined as a structural cardiac diagnosis expected to require surgery during the first year of life, who were admitted to the cardiac intensive care unit (CICU). Infants were required to be younger than 3 months of age at the time of their FOF, and to experience the FOF during their index hospitalization. Infants were excluded if they did not have sufficient medical record documentation to characterize cardiac diagnosis or feeding details.

### Variables

Exposure variables, hypothesized to impact nutrition type or route at the FOF based on clinical experience and previous literature, included [1] the presence of a parent at the FOF, defined as a primary caregiver [2], infant insurance type, defined as public (“US Public,” “US Medicaid,” or “US Medicare”) or private (“Private,” “Free Universal Health Care,” or “Self-pay/International”) [3], early infant age at the FOF, defined as <2 days old [4], the infant’s surgical status, defined as preoperative, postoperative, or non-surgical (includes catheter-based procedures), and [5] whether the infant had a feeding therapist or lactation consult before or during the FOF. Covariates for models were determined a priori and included insurance type, single ventricle cardiac physiology, infant age, and diagnosed or suspected genetic/chromosomal syndrome, as appropriate for the model. Preterm birth (<37 weeks) was considered as a covariate, but did not improve the models.

### Statistical analysis

Descriptive statistics (e.g., n, %) and data visualization characterized the type and route of nutrition used at the FOF. To explore patterns by infant age, the cohort was divided into approximate quartiles, with quartile 1 (Q1) including infants with the FOF < 2 days old, quartile 2 (Q2) 2–3 days old, quartile 3 (Q3) 4–13 days old, and quartile 4 (Q4) ≥ 14 days old. We fit unadjusted and adjusted logistic regression models to estimate associations between the parental and clinical variables of interest and human milk or BF at the FOF. As missing data were minimal (3% missing single ventricle physiology; 1% surgical status; <1% age at FOF) with no clear patterns of missingness, we used complete cases for models. We assessed multicollinearity by calculating the variance inflation factor for each covariate. Statistical significance was set at *p* < 0.05, and analyses were completed in R version 4.5.0.

## Results

**Fig. 1:**
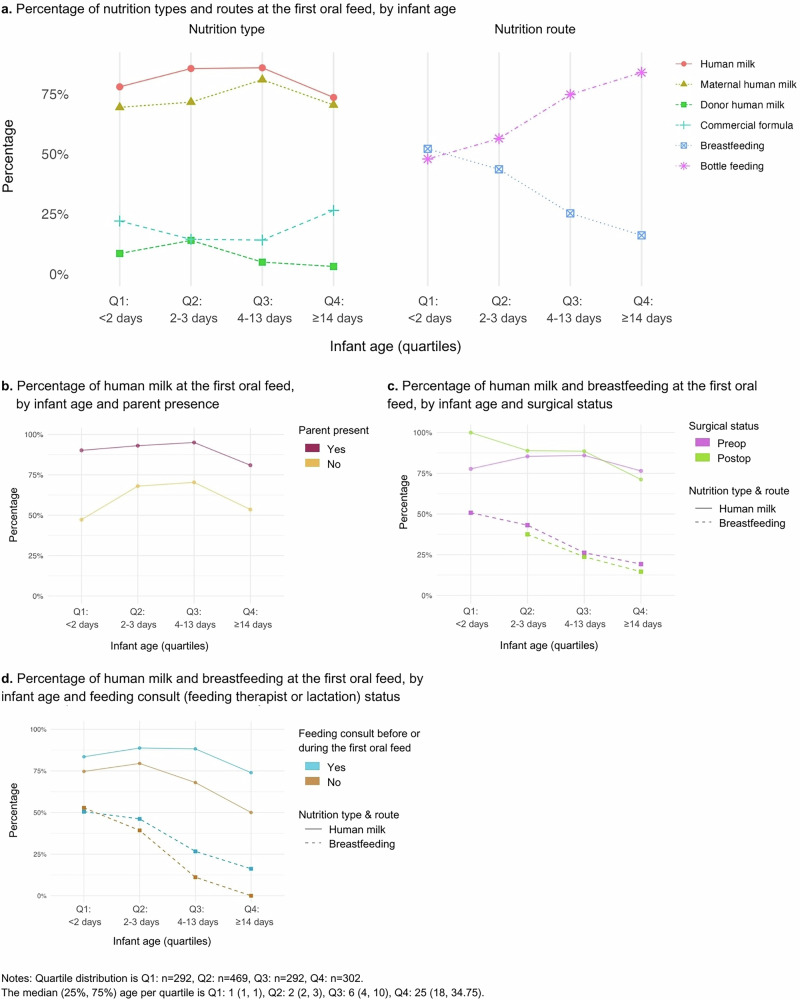
Feeding patterns at the first oral feed by infant age and clinical factors (*n* = 1355). **a** Percentage of nutrition types and routes at the first oral feed, by infant age. **b** Percentage of human milk at the first oral feed, by infant age and parent presence. **c** Percentage of human milk and breastfeeding at the first oral feed, by infant age and surgical status **d** Percentage of human milk and breastfeeding at the first oral feed, by infant age and feeding consult (feeding therapist or lactation) status.

Across 15 sites, 1355 infants had their FOF at a mean age of 10 days (Table 1), with a parent present for 73.4% of these feedings. Human milk (maternal or donor) was used in 78.5% of FOF events, and 34.5% of FOF events occurred via BF. Figure 1 illustrates the patterns of nutrition type and feeding route relative to the infant’s age. In this data visualization, human milk use appeared to be more frequent when a parent was present, a finding observed across all age quartiles. The lowest human milk prevalence (<50%) was observed among infants in the earliest FOF quartile (Q1) who did not have a parent present at the FOF. While BF and bottle feeding occurred with similar frequency for infants in Q1, the prevalence of BF at the FOF decreased over the first two postnatal weeks. Both human milk and BF occurred more frequently for infants with a feeding therapy or lactation consultation before or during the FOF.

**Table 1:** Sample characteristics (*N* = 1355).

	*n* (%) or mean (SD)
Sex	
Female	562 (41.5%)
Male	793 (58.5%)
Preterm	184 (13.6%)
(Unknown)	5
Insurance type	
Private	718 (58.9%)
Public	500 (41.1%)
(Unknown)	137
Interpreter needed	154 (11.4%)
(Unknown)	7
Parent primary language	
English	1187 (88.0%)
Spanish	112 (8.3%)
Another language	50 (3.7%)
(Unknown)	6
Primary cardiac diagnosis	
Single Ventricle	444 (33.8%)
Biventricular	869 (66.2%)
(Unknown)	42
Major genetic syndrome	
Yes/Suspected	331 (24.4%)
No/Unknown	1024 (75.6%)
Age (days) at FOF	10 (15)
Surgical status at FOF	
Preoperative	892 (67.8%)
Postoperative	364 (27.7%)
Other^a^	60 (4.6%)
(Unknown)	39
Parent present at FOF	878 (73.4%)
(Unknown)	159
Mother present at FOF	838 (70.3%)
If mother present, gave FOF	707 (90.5%)
Father present at FOF	377 (42.2%)
If father present, gave FOF	102 (30.7%)
FOF nutrition route	
Bottle	854 (63.0%)
Breastfeeding	468 (34.5%)
Other	33 (2.4%)
FOF nutrition type	
Maternal human milk	953 (70.3%)
Donor human milk	111 (8.2%)
Commercial formula	244 (18.0%)
Other	47 (3.5%)
Feeding consult before/during the FOF	963 (72.7%)
(Unknown)	31
Feeding therapist consult before/during the FOF	642 (47.9%)
(Unknown)	15
Lactation consult before/during the FOF	789 (60.6%)
(Unknown)	53

*FOF* first oral feed.

^a^“Other” includes cases in which surgery is likely in a subsequent admission, or when surgery is not likely to be needed.

Table 2 presents associations between key parental or clinical factors and human milk or BF at the FOF. In adjusted models, parent presence, private insurance, and a feeding/lactation consult were all associated with greater odds of human milk at the FOF, while private insurance, early age (<2 days) and preoperative or other/unknown surgical status was associated with higher likelihood of BF. Specifically, having a parent present was associated with 5.32 times greater odds of human milk use (95% CI: 3.86–7.35 times greater, *p* < 0.001). Infants who received a feeding or lactation consult were 2.49 times more likely to receive human milk at the FOF (1.81–3.43, *p* < 0.001). Infants with private insurance were 1.87 times more likely to receive human milk, (1.40–2.51, *p* < 0.001) and 1.82 times greater odds of BF at the FOF (1.40–2.36, *p* < 0.001), compared to infants with public insurance. Early age at the FOF was associated with 2.23 times higher BF likelihood (1.70–2.95, *p* < 0.001). Preoperative or other/unknown surgical status were associated with 3.66 and 3.16 times greater odds of BF, respectively (*p* < 0.001 for both).

**Table 2:** Associations between clinical and parental factors and human milk or direct breastfeeding at the first oral feed for infants with critical congenital heart disease (*n* = 1355).

Outcome: Human milk (maternal or donor) at the first oral feed			
	OR	95% CI	*p* value
**Model 1: Parent presence**			
a. Parent present (Unadjusted, *n* = 1196)	5.39	(3.98–7.32)	**<0.001**
b. Parent present (Adjusted, *n* = 1162)^a^	5.32	(3.86–7.35)	**<0.001**
**Model 2: Insurance type**			
a. Insurance type: Private (Unadjusted, *n* = 1218)	1.81	(1.37–2.39)	**<0.001**
b. Insurance type: Private (Adjusted, *n* = 1178)	1.87	(1.40–2.51)	**<0.001**
**Model 3: Early oral feeding**^**b**^			
a. Early oral feeding (Unadjusted, *n* = 1355)	0.77	(0.57–1.05)	0.098
b. Early oral feeding (Adjusted, *n* = 1313)	0.78	(0.57–1.07)	0.117
**Model 4: Surgical status**^**c**^			
a. Surgical status (Unadjusted, *n* = 1355)			
Preop	1.35	(1.00–1.79)	**0.045**
Other/Unknown	0.92	(0.56–1.55)	0.754
b. Surgical status (Adjusted, *n* = 1313)			
Preop	1.30	(0.96–1.75)	0.086
Other/Unknown	0.94	(0.56–1.63)	0.829
**Model 5: Feeding consult (therapist or lactation) before or during the first oral feed**			
a. Feeding consult occurred (Unadjusted, *n* = 1324)	1.77	(1.34–2.34)	**<0.001**
b. Feeding consult occurred (Adjusted, *n* = 1285)	2.49	(1.81–3.43)	**<0.001**

Outcome: Direct breastfeeding at the first oral feed			
	**OR**	**95% CI**	***p*** **value**
**Model 6: Insurance type**			
a. Insurance type: Private (Unadjusted, *n* = 1218)	1.91	(1.49–2.45)	**<0.001**
b. Insurance type: Private (Adjusted, *n* = 1178)	1.82	(1.40–2.36)	**<0.001**
**Model 7: Early oral feeding**			
a. Early oral feeding (Unadjusted, *n* = 1355)	2.39	(1.83–3.11)	**<0.001**
b. Early oral feeding (Adjusted, *n* = 1313)	2.23	(1.70–2.95)	**<0.001**
**Model 8: Surgical status**			
a. Surgical status (Unadjusted, *n* = 1355)			
Preop	3.19	(2.37–4.34)	**<0.001**
Other/Unknown	3.60	(2.22–5.83)	**<0.001**
b. Surgical status (Adjusted, *n* = 1313)			
Preop	3.66	(2.20–6.08)	**<0.001**
Other/Unknown	3.16	(2.33–4.33)	**<0.001**
**Model 9: Feeding consult (therapist or lactation) before or during the first oral feed**			
a. Feeding consult occurred (Unadjusted, *n* = 1324)	0.65	(0.51–0.83)	**<0.001**
b. Feeding consult occurred (Adjusted, *n* = 1285)	0.99	(0.75–1.31)	0.940

Bold = significant at *p* < 0.05

^a^All adjusted models were adjusted for insurance type (except models 2 and 6), single ventricle cardiac diagnosis, genetic/chromosomal syndrome, and infant age (except models 3 and 7).

^b^Early oral feeding is defined as <2 days old.

^c^The reference category for models 4 and 8 (surgical status) is postoperative.

Most infants (72.7%) received at least one feeding consult before or during the FOF. A total of 468 (35.5%) infants had consults from both a feeding therapist and lactation consultant, while 321 (24.2%) infants received a lactation consult only and 174 (12.9%) met with a feeding therapist only. In subgroup analysis (Table 3), the type of feeding consult was significantly associated with human milk at the FOF. Infants with a lactation consult only or both lactation and feeding therapist consults were approximately twice as likely to receive human milk at the FOF (2.21 and 2.03 greater odds, respectively; *p* < 0.001). In contrast, infants with only a feeding therapist consult were 47% less likely to receive human milk (95% CI: 23–63% lower odds, *p* < 0.001). Feeding consult type was not significantly associated with BF at the FOF.

**Table 3:** Associations between type of feeding consult and human milk or direct breastfeeding at the first oral feed for infants with critical congenital heart disease.

Outcome: Human milk (maternal or donor) at the first oral feed			
	OR	95% CI	*p* value
**Model 1: Both feeding therapist and lactation consult**			
a. Unadjusted (*n* = 1363)	1.31	(0.99–1.75)	0.059
b. Adjusted (*n* = 1276)	2.03	(1.46–2.84)	**<0.001**
**Model 2: Feeding therapist consult only**			
a. Unadjusted (*n* = 1347)	0.52	(0.37–0.74)	**<0.001**
b. Adjusted (*n* = 1305)	0.53	(0.37–0.77)	**0.001**
**Model 3: Lactation consult only**			
a. Unadjusted (*n* = 1324)	2.64	(1.83–3.92)	**<0.001**
b. Adjusted (*n* = 1285)	2.21	(1.51–3.30)	**<0.001**
**Outcome: Breastfeeding at the first oral feed**			
	**OR**	**95% CI**	***p*** **value**
**Model 4: Both feeding therapist and lactation consult**			
a. Unadjusted (*n* = 1363)	0.60	(0.47–0.77)	**<0.001**
b. Adjusted (*n* = 1276)	0.89	(0.67–1.18)	0.430
**Model 5: Feeding therapist consult only**			
a. Unadjusted (*n* = 1347)	0.84	(0.59–1.18)	0.321
b. Adjusted (*n* = 1305)	0.97	(0.67–1.38)	0.869
**Model 6: Lactation consult only**			
a. Unadjusted (*n* = 1324)	1.24	(0.95–1.61)	0.107
b. Adjusted (*n* = 1285)	1.11	(0.84–1.46)	0.470

Bold = significant at *p* < 0.05

Adjusted models were adjusted for insurance type, single ventricle cardiac diagnosis, genetic/chromosomal syndrome, and infant age.

## Discussion

In this large, multisite cohort we described, for the first time, the patterns and prevalence of human milk and BF at the FOF among infants with CCHD. Across 15 sites, fewer than 80% of infants received human milk at the FOF and only 35% were breastfed. While direct comparison to healthy infants is difficult due to a lack of differentiation between human milk feeding and direct BF in national and global reports, the overall human milk and BF rates in our cohort are lower than the 85.7% of US infants “ever breastfed” in 2022, with direct BF particularly low [26]. Evidence on FOF experiences for hospitalized infants is primarily limited to single-site studies of preterm infants [19–21, 24], with wide variation in reported FOF practices. Pineda et al. found that only 16.4% of infants who initiated enteral human milk feeding (*n* = 66) were breastfed at the FOF [19], while Casey et al. reported 75% BF at the FOF for infants whose parent intended to breastfeed (*n* = 69) [24]. Similar to our results, Suberi et al. found that 40% of very low birth weight infants (*n* = 255) received their FOF via BF, in a cohort that was not limited to infants whose parent intended to provide human milk or BF [20]. The only known previous study on first feeding experiences for newborns undergoing surgery, including infants with CCHD (*n* = 24), reported that 69.5% of infants received human milk for their first enteral feed; however, this study did not investigate the FOF [23]. Thus, our findings address a critical knowledge gap by establishing baseline data on the timing, type, and route of nutrition at the FOF for infants with CCHD, providing a foundation for future research and QI initiatives aimed at optimizing early feeding practices in this high-risk population.

Parent presence emerged as a strong predictor of human milk use at the FOF. Strikingly, fewer than half of the youngest infants (0 or 1 day old) without a parent present received human milk at their FOF. This finding highlights a critical window for intervention, particularly as early formula exposure is consistently associated with early cessation of human milk and BF in both hospitalized and healthy newborns [16, 17, 23]. Furthermore, in high-risk populations, human milk (particularly maternal human milk) is associated with lower rates of infection, necrotizing enterocolitis, and shorter hospital stays compared with formula, and is therefore considered the preferred enteral nutrition when available [10, 12, 13]. Accordingly, current practice recommendations increasingly frame human milk as the preferred enteral nutrition source for medically fragile infants, with formula reserved for situations in which maternal or donor milk is unavailable or contraindicated [9, 10, 27].

Beyond nutritional and clinical implications, early feeding is also a core relational experience. For medically fragile infants, the FOF may be one of the first opportunities for parents to participate in attuned caregiving. These early feeding moments situate the infant in the natural parent niche, a caregiving environment defined by closeness, attunement, and co-regulatory support. Parental presence, gentle physical contact, and responsive engagement during feeding support early co-regulation and help establish a sense of safety and connection, processes foundational for emerging attachment relationships [28]. When parents are absent at this early feeding moment, their ability to assume a confident caregiving role may be disrupted, particularly in the context of the stress and separation inherent to CICU hospitalization [7].

Given the clear association between parent presence and human milk use at the FOF, targeted strategies are needed to support families and strengthen early feeding practices. Potential strategies for practice improvement include ensuring donor human milk access as a bridge to maternal milk [29], enhancing clinician education about the potential long-term impact of the FOF [30–32], incorporating prenatal discussions about the FOF to document parent feeding preferences and plan for early colostrum and human milk use [32], and prioritizing parent involvement in the FOF [33]. Notably, donor human milk is widely accepted as standard of care in NICUs due to decades of evidence linking human milk to reduced risk of necrotizing enterocolitis [34], and 88% of level 3 and 4 US NICUs reported active donor human milk programs in 2020 [35]. In contrast, our cohort demonstrated low donor human milk use, with only 8.5% of infants in Q1 (<2 days) and 8.2% overall receiving donor milk at the FOF, highlighting an opportunity for practice change to improve access to human milk in the context of early parent-infant separation. While there is little evidence specifically focused on donor human milk for CHD populations, the American Academy of Pediatrics recommends donor human milk for high-risk infants [36], and our results highlight an opportunity for institutions to improve processes for donor milk access, consent, and prioritization.

Breastfeeding at the FOF was uncommon in our cohort and declined over the first 2 postnatal weeks. Early infant age and, relatedly, preoperative status were both associated with a higher likelihood of BF. These findings suggest a need for early strategies to preserve later BF opportunity, particularly since there is a finite developmental window for BF establishment [32]. Evidence-based approaches include enhanced lactation support, as specialized staff training and lactation services have been associated with higher BF establishment and duration for NICU populations [30, 37, 38]. Early and frequent skin-to-skin (STS) care increases BF initiation [39, 40] and success and is safe and feasible for infants with CCHD even in the immediate postnatal period [41–44]. Non-nutritive latching when the infant is not able to take feeds by mouth has been shown to increase BF for preterm infants [45]. Additionally, in a cohort of newborns undergoing CCHD surgery, increasing frequency of oral care with human milk during the first 7 postnatal days was associated with substantially greater odds of BF at discharge [15]. Future areas for investigation include development of clinical guidelines to support BF at the FOF postoperatively, when there may be increased concern for aspiration or a heightened focus on volume intake [32].

While infants who received some form of feeding consult (feeding therapist and/or lactation) before or during the FOF had higher rates of human milk use, this result appeared to be driven largely by lactation consultants, with feeding therapist consults negatively associated with human milk in the absence of lactation involvement. Notably, neither feeding therapist consults nor lactation consults were linked to higher BF rates. Several factors may explain these patterns. Parents who did not intend to provide human milk or BF may have been more likely to receive feeding therapy without lactation support; however, in this high-risk population, donor milk use may still be warranted. Additionally, there is often limited BF-specific training among feeding therapists. As Mahurin-Smith and Genna note, few speech-language pathologists receive BF training and may have limited understanding of the physiology of BF compared to bottle-feeding, constraining their assessments and recommendations to bottle-based approaches [46]. Importantly, BF and human milk content is currently absent from the American Speech-Language-Hearing Association standards for speech-language-pathologist curriculum, representing an actionable gap [47, 48]. Targeted, BF-specific education for feeding therapists may be important in enhancing their ability to provide lactation-supportive care in the context of CCHD.

Finally, our finding that public insurance was associated with lower rates of human milk use and BF at the FOF suggests that social drivers of health influence lactation outcomes. This pattern is consistent with evidence from both term [49] and preterm [50] populations and aligns with studies in CCHD, where insurance type has been shown to be a strong predictor of human milk use and BF at hospital discharge in a national cohort of infants with single ventricle CHD [22]. Because early feeding experiences help shape parental role confidence, bonding, and the development of secure attachment, disparities in parent presence and human milk access at the FOF may compound existing social and structural inequities. Ensuring that families facing socioeconomic barriers receive proactive support may therefore be essential not only for improving nutrition outcomes but also for fostering equitable early relational experiences.

This study has several limitations. Because the data were collected as part of a QI initiative, parent presence at the FOF may have varied over time. Some sites reported difficulty in tracking the FOF, which may have led to underreporting of infants without a feeding consult before or during the FOF. Reporting may have been impacted by differences across sites in FOF electronic health record documentation practices. The retrospective design precludes causal inference, and available covariates were limited to those collected as part of the QI project. For example, we lacked information about parental characteristics such as maternal intention to provide human milk or BF. While the multi-site design is a strength, sites participating in the QI project may be different than non-participating sites; thus, our results may not be fully generalizable. Future research is needed to identify institutional and cultural barriers to human milk and BF at the FOF, and to investigate the potential impact of the FOF on long-term nutrition outcomes for children with CCHD.

In conclusion, this large, multisite study provides the first description of the prevalence and patterns of human milk and BF at the FOF among infants with CCHD, identifying substantial gaps compared to the general US population. Parent presence and lactation support emerged as potentially modifiable factors associated with human milk at the FOF, and early oral feeding practices in the absence of a parent may be a particularly critical target for focused intervention. Early/preoperative oral feeding was associated with increased BF, suggesting a need for intentional strategies to support later BF opportunity. Infant insurance type was associated with both human milk and BF, reinforcing the impact of social drivers of health on lactation outcomes for hospitalized infants. Recommendations for clinical practice include improving institutional donor human milk access, offering specialized clinician education, prioritizing parent presence at the FOF, and implementing evidence-based practices that facilitate direct BF (e.g., skin-to-skin care, non-nutritive latching, oral immune therapy with human milk). Taken together, the findings from this study highlight an opportunity to optimize infant nutrition and strengthen the parent-infant connection during a period of heightened vulnerability, through intentional strategies to increase human milk and BF at the FOF for infants with CCHD.

## Data Availability

Due to patient privacy regulations and institutional data-use restrictions, the data for this study cannot be made publicly available and cannot be shared.
